# Can COVID-19 pandemic boost the epidemic of neurodegenerative diseases?

**DOI:** 10.1186/s13062-020-00282-3

**Published:** 2020-11-27

**Authors:** Alexei Verkhratsky, Qing Li, Sonia Melino, Gerry Melino, Yufang Shi

**Affiliations:** 1 Faculty of Biology, Medicine and Health, The University of Manchester, Manchester, M13 9PT UK; 2 Achucarro Center for Neuroscience, IKERBASQUE, 48011 Bilbao, Spain; 3 CAS Key Laboratory of Tissue Microenvironment and Tumor, Shanghai Institute of Nutrition and Health, Chinese Academy of Sciences, 320 Yueyang Road, Shanghai, 200031 China; 4 University of Rome Tor Vergata, via Cracovia 1, 00133 Rome, Italy; 5 State Key Laboratory of Radiation Medicine and Protection, The First Affiliated Hospital of Soochow University, Institutes for Translational Medicine, Soochow University Medical College, Suzhou, 215123 Jiangsu China

**Keywords:** SARS-Cov-2, COVID-19, Systemic inflammation, Brain, Cognitive deficits, neurodegeneration

## Abstract

The pandemic of Coronavirus Disease 2019 (COVID-19) presents the world with the medical challenge associated with multifactorial nature of this pathology. Indeed COVID-19 affects several organs and systems and presents diversified clinical picture. COVID-19 affects the brain in many ways including direct infection of neural cells with SARS-CoV-2, severe systemic inflammation which floods the brain with pro-inflammatory agents thus damaging nervous cells, global brain ischaemia linked to a respiratory failure, thromboembolic strokes related to increased intravascular clotting and severe psychological stress. Often the COVID-19 is manifested by neurological and neuropsychiatric symptoms that include dizziness, disturbed sleep, cognitive deficits, delirium, hallucinations and depression. All these indicate the damage to the nervous tissue which may substantially increase the incidence of neurodegenerative diseases and promote dementia.

The second wave of COVID-19 pandemic engulfs the world with number of people infected with SARS-Cov-2 raised over 56 millions with virus claiming more than 1.4 millions of lives [1–4]. These numbers are *au pare* with another epidemic that slowly but certainly swamps the world – the epidemic (as it was defined by Robert Katzman in 1976 [5]) of neurodegenerative diseases, which propagate through our rapidly ageing population. Along with the rapid changes in living environment and lifestyles, the number of people suffering from neurodegenerative disorders, that include vascular dementia, Alzheimer’s and Parkinson disease, frontotemporal dementia, various tauopathies and so forth are estimated at 60–70 millions worldwide and these numbers are rising with projection of doubling within next 20 years [6–8]. The numbers of death from AD alone increased by 146% between year 2000 and year 2018 [9]. Although the main battlefield of neurodegenerative disorders is the brain, these diseases are title connected with the overall body state, with the onset and course of neurodegenerative diseases being substantially affected by lifestyle and somatic pathologies. Major peripheral disease, including trauma, sepsis, gastrointestinal disorders, kidney pathologies metabolic abnormalities and infections associated with systemic inflammation exacerbate the evolution of neurodegeneration [10–13]. Furthermore neurodegenerative disorders are linked to psychological stress, sleep disturbances, and to depression, all of which may accelerate the onset and evolution of neurodegeneration. The COVID-19 being generally manifested as a viral pneumonia with respiratory distress and prominent systemic inflammation is likely to modify the course of neurodegenerative pathologies. One common feature shared by neurodegeneration and COVID-19 is age: age is the major risk factor for neurodegenerative diseases [14], while old patients infected with SARS-Cov-2 present the most severe clinical picture with prolonged course of the disease [15].

The neurological and psychiatric complications of COVID-19 are widely reported; these include encephalitis, cerebral infarction, delirium, depression, delirium, psychosis Guillain-Barré syndrome [16–20], Miller-Fisher syndrome, and etcetera [19, 21–28]. In at least three cases the COVID-19 brought with it symptoms of clinical Parkinsonism demonstrating therefore a potential direct link between the SARS-CoV-2 infection and neurodegeneration (see [29] for details). These acute parkinsonian symptoms may be related to an acute damage to the dopaminergic system being thus distinct from sporadic classical Parkinson disease, and yet such association required serious consideration. How COVID-19 may affect the neurodegenerative process and what are the underlying mechanisms? Below we shall try to overview several possible scenarios (Fig. 1).

(i) *Direct infection of neural cells with SARS-Cov-2.*

**Fig. 1:**
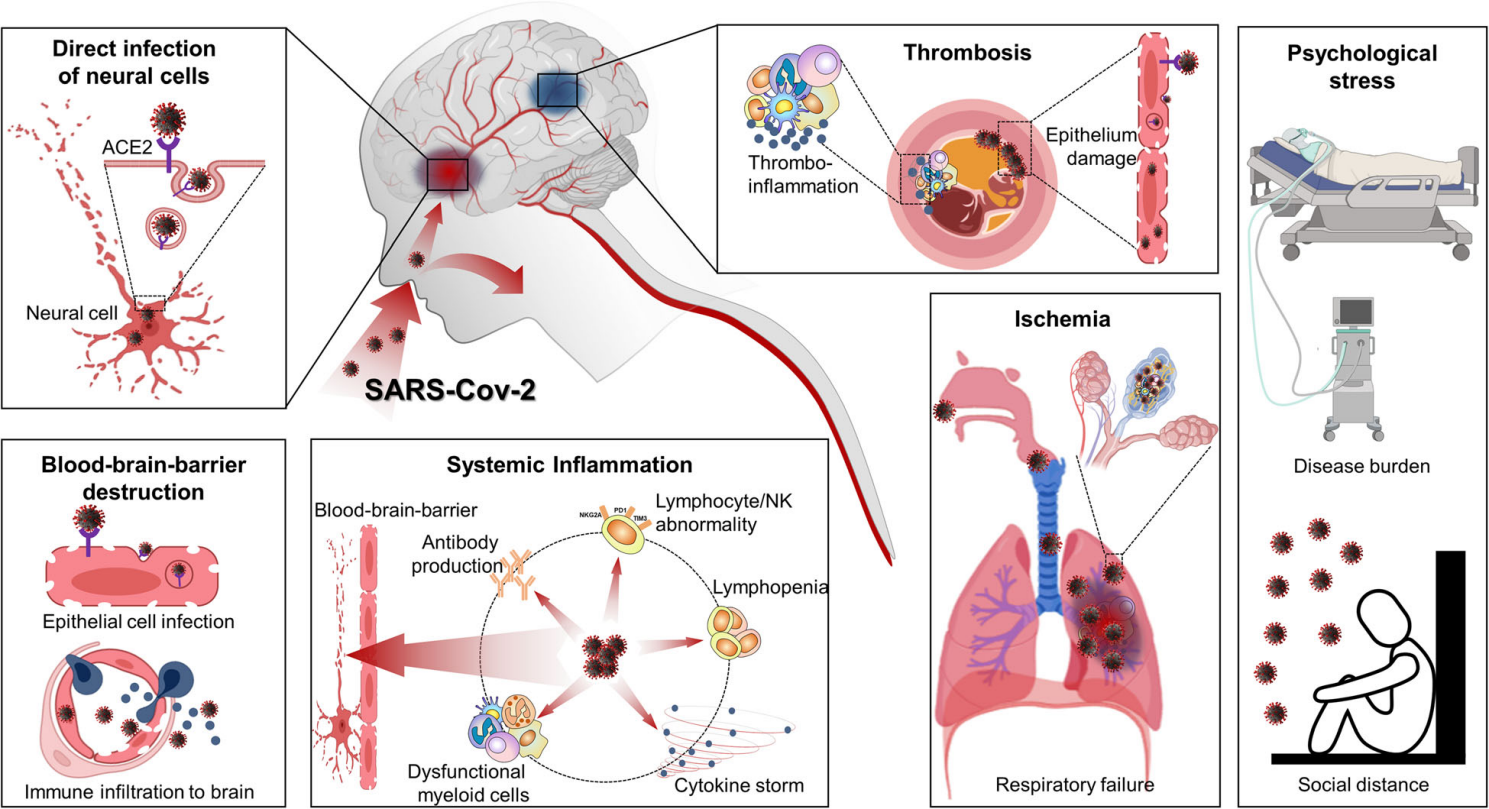
COVID-19 damages the brain: possible links to neurodegeneration. See text for explanations and details

The common way of the SARS-Cov-2 virus into the cell proceeds through binding of the RBD domain of the S-protein upon cleavage by furin to the receptor angiotensin-converting enzyme 2 (ACE2) with subsequent internalisation of the virus probably by endocytosis in either clathrin- or pH-dependent manner, which may also involve endosomal proton pump and NAADP-sensitive intracellular two-pore channel 2 [30–33]. The angiotensin system is operational in the nervous tissue and many cells of the brain including neurones and neuroglia express its components including ACE2 and furin [34–36]. In particular, ACE2 expressing neural cells are located in the brain stem, in the circumventricular organs (CVOs), the subfornical organ, paraventricular nucleus (PVN), nucleus of the tractus solitarius (NTS), and rostral ventrolateral medulla, all these structures having high vascularisation and physiologically leaky blood-brain barrier [37], which permits direct contact with blood-borne viral particles. An alternative pathways for SARS-CoV-2 entry into the brain through nasal epithelium with subsequent retrograde and trans-synaptic penetration through axons of olfactory neurones; this may bring the virus into the olfactory bulb [38, 39].

The virulence of SARS-CoV-2 may also involve neuropilin-1, known to bind furin-cleaved substrates [40, 41]. It appeared that spike coronavirus protein shows a polybasic Arg-Arg-Ala-Arg carboxyl-terminal sequence on the cleaved fragment of S1 that matches the predicted C-end rule (CendR) motif for physical interaction with neuropilins. The structure of neuropilin-1 has been resolved and the coordination of the extracellular domains 1–4 (a1a2b1b2) is shown in Fig. 2 [42]. It tunes out that the domain B1 is able to bind the small inhibitor molecule EG00229 [43], which suppresses the infectivity of SARS-CoV-2 [41]. Similarly, monoclonal antibody against neuropilin-1 significantly reduces viral infectivity [40]. In the latter case, post-mortem autopsies of the olfactory neuronal detected neuropilin-1 at the entry site for the virus. These data offer a significant potential intervention pathway for the treatment of the infection, including its involvement of the central nervous system [44]. Prediction of clinical outcome is essential for medical practice [45–53]; unfortunately, pathobiology of COVID-19 is still missing identifiable molecular determinants of disease progression and clinical outcome.

**Fig. 2:**
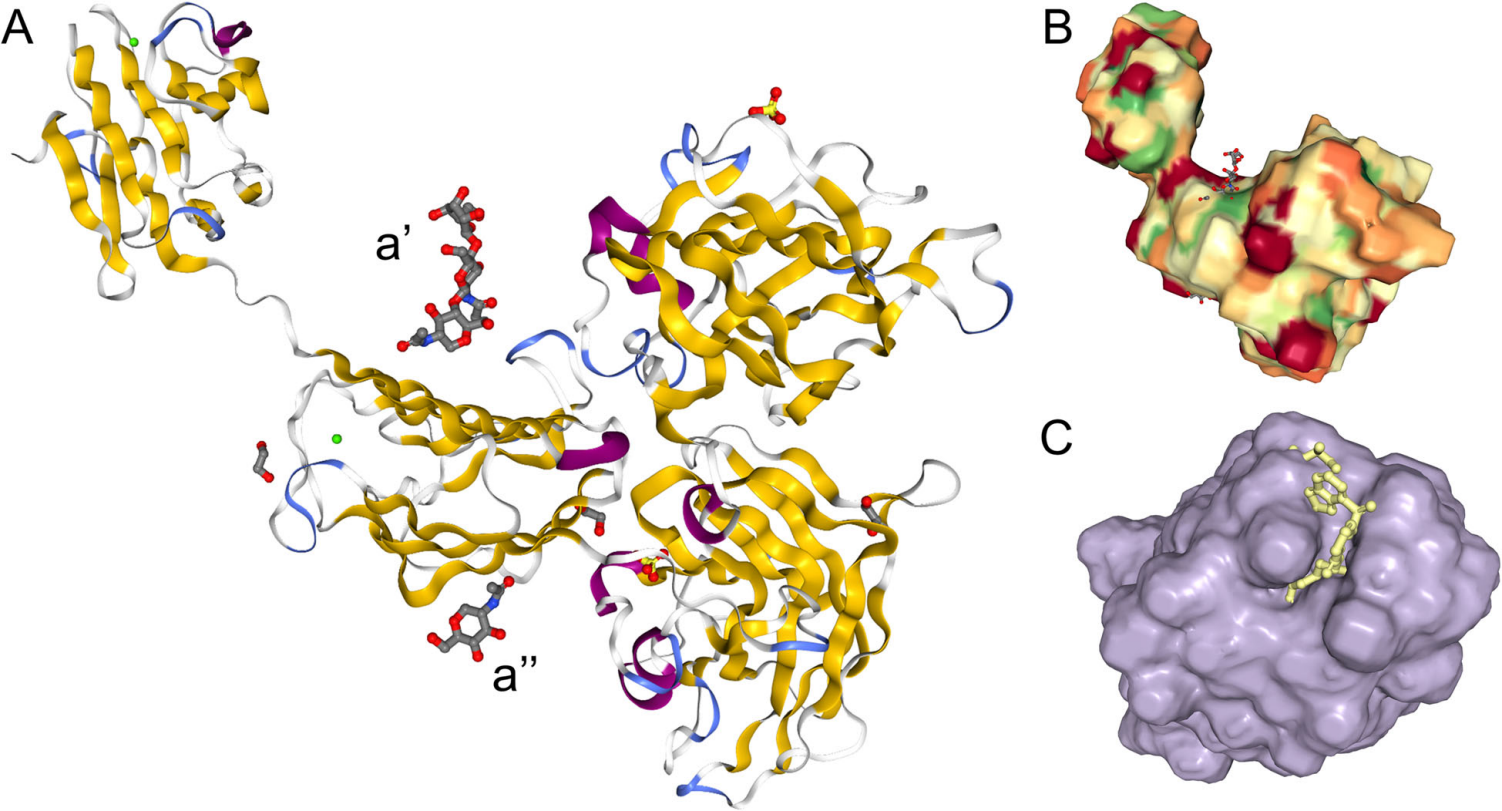
Structural constrains of neuropilin-1. **a**. Secondary structure of the extracellular domains 1–4 (a1a2b1b2) of mouse Neuropilin-1, PDB assignment 4GZ9 (DOI: 10.2210/pdb4GZ9/pdb) [42]. α’- β-D-mannopyranose-(1–4)-2-acetamido-2-deoxy-β-D-glucopyranose-(1–4)-2-acetamido-2-deoxy-beta-Dglucopyranose. a’’ = 1,2-ethanediol. **b**. Surface structure of neuropilin-1 shown by hydrophobicity (same source of panel A). **c**. Binding of the small inhibitor molecule EG00229 on the B1 domain of human neuropilin-1. PDB assignment 3I97 (DOI: 10.2210/pdb3I97/pdb) [43]

Infection of neurones and neuroglial cells have been documented in vitro, in particular in 2D cultures and brain organoids derived from human stem cells [54–57]. The viral particles have been also found in the post-mortem brain tissues obtained from COVID-19 victims; the viral load was found in 30–50% of all specimens [58, 59]. Can these viruses damage neural cells beyond repair and instigate neurodegenerative process? This is difficult to assess at the moment; it is known that influenza can be associated (rarely) with encephalitis leading to a substantial damage to the brain tissue [60]. This damage has been detected at the cellular level; in particular such damage was manifested by clasmatodenrosis, indicative of severe degeneration of astrocytes [61]. Hitherto, such pronounced degenerative change in the SARS-CoV-2 infected brains has not been characterised. In addition, the persistence of SARS-CoV-2 in the brain after viral clearance from the respiratory system and blood remains to be characterised.

(ii) *Systemic inflammation.*

The systemic inflammation is the main feature of severe cases of COVID-19; the “cytokine storm” reflecting massive increase of pro-inflammatory factors in the blood, is a singular feature of COVID-19 pathogenesis [62]. It should be noted that in severe COVID-19 patients, T cells are often lost and the inflammation is characterized by innate immune responses [63]. How innate cytokines affect the central nervous system in absence of adaptive cytokines is not clear. The link between systemic inflammation and neurological as well as neuropsychiatric diseases is universally acknowledged with both innate and adaptive immune responses affecting the brain [64–67]. Cytokines, chemokines or even activated blood-borne immune cells can enter the brain through subfornical organs; in addition cytokines can compromise the BBB thus opening an alternative entry route for pro-inflammatory agents [68]. Even at low intensity of systemic inflammation, invasion of pro-inflammatory factors initiates sickness behaviour, a wide-spread syndrome characterised by depressive-like behaviours, lack of appetite, general fatigue, abnormal sleep patterns and decreased cognitive agility [69, 70]. Conceptually sickness behaviour can be regarded as systemic defensive response aimed at conserving energetic responses for fighting the infection. Nonetheless, the immunological pathways activated during sickness behaviour are potentially damaging being linked to pathogenesis of major depression and neurodegeneration [71].

In sever cases systemic inflammation causes acute brain damage associated with psychiatric symptoms as well as with cognitive impairments indicative of neurodegeneration. In sepsis with bacteraemia, which is paramount example of severe systemic inflammation, almost 80% of patients develop sepsis-associated encephalopathy [72, 73] and sepsis-associated delirium [74, 75]. In the elderly, the sepsis associated encephalopathy often instigates severe cognitive impairments, both acute and long-lasting and exacerbates exiting neurodegenerative pathology [68, 76]. Severe COBID-10 triggers systemic inflammation most likely on a comparable scale with sepsis [77], which thus may result in similar deleterious cognitive outcomes and is likely to aggravate existing neurodegenerative pathologies.

The first line of defence of the CNS against systemic inflammation and systemic infection is formed by astrocytes and microglia; both are participating towards *glia limitans* that provides parenchymal part of blood-brain barrier formed by astroglial endfeet and processes of juxtavascular microglia [78, 79]. Systemic inflammation and associated disruption of BBB, which allows infiltration of various damage-associated molecular patterns into the nervous tissue, triggers reactive astrogliosis [80] and reactive microgliosis [81, 82]. Reactive gliosis is a powerful and evolutionary conserved defensive mechanism; suppression of gliotic response exacerbates neuropathologies including those triggered by infectious lesions [83, 84]. Both glial responses are present in the COVID-10 affected brains [85] with numerous documentations for areas of gliosis in COVID-19 post-mortem brains [86, 87]. At the same time systemic inflammation may damage glial cells, resulting in their atrophy and loss of functions; dystrophic astrocytes and microglia are known to facilitate initiation and pathological development of neurodegenerative disorders [88–91].

It has been also realised that COVID-19 patients with basic metabolic disorders such as type II diabetes are prone to develop severe inflammation. COVID-19 infection has been shown to cause hyperglycaemia, thus stimulating glycolysis [92] which, in turn, pushes macrophages to the pro-inflammatory phenotype [93], that may predispose patients to severe COVID-19 presentation with increased lethality. It remains unknown how long the hyperlycaemia last and how stable the pro-inflammatory macrophage maintain, although the longer the pro-inflammation persist, the more effect it will be on the development of neurodegeneration. Some COVID-19 patients maintain positive test for virus in stool months after viral negativity in the nose and in the throat [94]. Of note, the S-protein possesses a sequence similar to well known super-antigen staphylococcal enterotoxin B (SEB) [95]. This superantigen is linked to a persistent gut inflammation and impaired gut microbiota which represent another factor contributing to the development of neurodegenerative diseases.

(iii) *Autoimmunity in COVID-19 associated brain damage.*

Another brain-damaging mechanism, linked to systemic infection is associated with autoimmunity. Indeed autoimmune attack is known to cause encepahalopathies with neurological and psychotic symptoms [96–98]; and sometimes the acute psychosis is the leading symptoms, as, for example, in the case of anti-N-methyl-D-aspartate (NMDA) receptor encephalitis [99, 100]. Viral infections have been shown to induce autoimmunity through, for example, a phenomenon known as molecular mimicry [101, 102]. In the context of COVID-19 production of antiphospholipid autoantibodies has been detected [103] The brain being an immunoprivileged tissue protected by the BBB is particularly vulnerable for an autoimmune attack. The autoimmune damages to the CNS in particular may affect the white matter tracts and peripheral nerves [104]; the latter type of damage presented as a Guillain-Barré syndrome [17, 19], or its cranial variant Miller-Fisher syndrome [28] have both been reported in COVID-19 sufferers. Nonetheless such cases remain rare, and no autoantibodies have been ever identified in the cerebrospinal fluids of COVID-19 patients.

(iv) *Ischaemia.*

The main clinical presentation of COVID-19 is malignant pneumonia causing, even in mild cases, decrease in blood oxygenation; in severe cases omnipresent inflammation of lung tissue is associated with profound respiratory failure and severe hypoxia. Such global hypoxia inevitably affects the brain, the organ with highest demand for oxygen that is needed to sustain energy-hungry nervous tissue. Cerebral hypoxia has multiple negative effects on the brain. The primary damage is associated with mounting of respiratory alkalosis and energy deprivation; decrease of arterial oxygen saturation below 75% causes profound impairments of neuronal activity [105]. Hypoxia also causes oxidative damage to neural cells due to a rapid increase in production of reactive oxygen species, which swiftly overpower rather limited brain antioxidative defences [106]. Brain hypoxia is also directly linked to activation or exacerbation of inflammatory response by stimulating hypoxia inducible factors and the NF-κB signalling cascade, which in turn prompt the release of pro-inflammatory factors [107]. In summary, severe and/or prolonged hypoxia may cause widespread damage to brain structures being thus directly linked to neurodegeneration and cognitive deficits.

(v) *Thrombosis and stroke.*

The systemic inflammation accompanying COVID-19 increases blood levels of fibronectin, arguably through stimulating its liver synthesis [108]. Increased fibronectin facilitates clot formation and 20–50% of COVID patients demonstrate thrombotic and thromboembolic complications [109, 110]. Among these complications stroke has been described relatively frequently with numbers varying between 1.6% and up to 20% of hospitalised patients [111–113], including people of young ages [114, 115]. The link between stroke and neurodegeneration is well documented. Stroke is associated with stroke-induced secondary neurodegeneration [116, 117] as well as with increased risk of Alzheimer’s disease [118]. Covid-19 associated thrombosis therefore can be directly linked to neurodegenerative diseases.

(vi) *Psychological stress.*

Patients hospitalised with severe forms of COVID-19 are exposed to a prolonged and malignant stress associated with the gravity of their conditions, with extended period lung ventilation, with grave atmosphere of the intensive care unit and with periods of delirium, unconsciousness and, sometimes, coma. This aversive experience amounts to the trauma likely to induce the post-traumatic stress disorder, which is also linked to immune pathology [119, 120]. In addition, maladaptive stress response (linked to powerful and long lasting stressors) exacerbates both systemic inflammation and inflammatory damage to the brain through activation of the hypothalamic-pituitary-adrenal axis with increase in gluocorticoids. Neuroinflammation is deeply associated with several neuropsychiatric and neuro-cognitive diseases, including depression, psychosis and neurodegeneration [121–124]. Previous analysis of SARS-Cov-1 infection revealed alarmingly high prevalence of neuropsychiatric sequalae with 40% of patients suffering from post-traumatic stress disorder and 36% form depression in 50–80 months after their hospitalisation [125]. Psychological stress affects not only COVID-19 patients but also general population due to lockdown, self isolation and fear; these factors are especially prominent between old people. Depression is a well known risk factor of dementia and psychological burden of COVID-19 may increase the rate of neurodegenerative diseases in the aftermath of the pandemic [126].

## Recapitulation

The pandemic of Coronavirus Disease 2019 (COVID-19) presents the world with the medical challenge associated with multifactorial nature of this pathology. COVID-19 affects the brain in many ways; often the COVID-19 is manifested by neurological and neuropsychiatric symptoms that include dizziness, disturbed sleep, cognitive deficits, delirium, hallucinations and depression. All these signal the damage to the nervous tissue which may substantially increase the incidence of neurodegenerative diseases and promote dementia.

## Data Availability

Not applicable.
